# Inflammatory Hot Phases in Arrhythmogenic Cardiomyopathy: A Dynamic Electrophysiologic Substrate Beyond Scar

**DOI:** 10.1016/j.jacadv.2026.103154

**Published:** 2026-08-22

**Authors:** Mehrdad Zarghami, Neda Dianati Maleki, Sajieh Naddaf, Tarek Abdulhadi, Magdalena Grabos, Ali Kanan, DinMukhammed Osser, Azadeh Mahdavinia, Shreya Gulati, Abhijeet Singh

**Affiliations:** a Department of Internal Medicine, Jamaica Hospital Medical Center, Queens, New York, USA; b Thrombosis Research Group, Brigham and Women's Hospital, Harvard Medical School, Boston, Massachusetts, USA; c Division of Cardiology, Cardiac Imaging, Stony Brook University Hospital, Stony Brook, New York, USA; d Department of Internal Medicine, Danbury Hospital / Zucker School of Medicine at Hofstra/Northwell, Danbury, Connecticut, USA; e Biochemistry and Structural Biology Graduate Program, Stony Brook University, Stony Brook, New York, USA; f Division of Cardiology, Electrophysiology Section, Stony Brook University Hospital, Stony Brook, New York, USA

**Keywords:** arrhythmogenic cardiomyopathy, cardiac MRI, hot phase, NLRP3 inflammasome, ventricular tachycardia

## Abstract

Arrhythmogenic cardiomyopathy (ACM) has traditionally been conceptualized as a progressive inherited cardiomyopathy characterized by fibrofatty replacement and ventricular arrhythmias. Emerging clinical and translational evidence suggests that selected patients experience episodic inflammatory exacerbations, or “hot phases,” that mimic acute myocarditis and may accelerate myocardial injury, scar formation, and arrhythmic vulnerability. Proposed mechanisms include desmosomal instability, maladaptive mechanotransduction, inflammasome activation, cytokine signaling, and humoral immune reactivity, including anti-desmoglein-2 responses. Cardiac magnetic resonance tissue characterization, particularly T2 mapping combined with late gadolinium enhancement, may help distinguish active edema from chronic scar and support genotype-informed phenotyping. This review synthesizes mechanistic, imaging, genetic, and electrophysiologic evidence for the ACM hot-phase paradigm and proposes a structured approach to evaluation. At present, suspected hot phase should prompt exclusion of mimics, rhythm surveillance, genotype assessment, family screening, and standard ACM risk-based care, whereas immunomodulatory therapy remains investigational or context-dependent.

## Highlights

• Arrhythmogenic cardiomyopathy (ACM) hot phases may represent episodic inflammatory injury superimposed on a genetically determined arrhythmogenic substrate, often mimicking acute myocarditis.
• Desmosomal dysfunction may promote mechanical injury, maladaptive mechanotransduction, inflammasome activation, cytokine signaling, and anti-desmoglein-2 immune reactivity in selected phenotypes.
• Anti-desmoglein-2 reactivity and innate immune activation may reflect or amplify myocardial injury, but their pathogenic roles remain incompletely established.
• Cardiac magnetic resonance T2 mapping with late gadolinium enhancement assessment may help distinguish active edema from chronic scar during myocarditis-like ACM presentations.
• Suspected ACM hot phase should trigger structured phenotyping, rhythm monitoring, genotype assessment, family screening, and standard ACM risk-based management; immunomodulation remains investigational or context-dependent.

Arrhythmogenic cardiomyopathy (ACM) is an inherited myocardial disorder characterized by ventricular arrhythmias (VAs), progressive structural remodeling, and heightened risk of sudden cardiac death. Historically described as arrhythmogenic right ventricular (RV) dysplasia/cardiomyopathy, the disease was initially conceptualized as a predominantly right-sided disorder driven by progressive fibro-fatty myocardial replacement.^1^^,^^2^ Contemporary understanding, however, recognizes ACM as a broader phenotypic spectrum frequently involving biventricular or left-dominant disease manifestations, prompting adoption of updated diagnostic frameworks and nomenclature.^2^, ^3^, ^4^, ^5^, ^6^ Traditional models of ACM pathogenesis describe a gradual and largely irreversible progression from desmosomal dysfunction to structural myocardial degeneration and scar formation.^1^ Yet this framework may incompletely explain the increasingly recognized phenomenon of acute myocarditis–like presentations observed in subsets of patients with ACM, particularly those harboring desmoplakin (DSP) and other nonclassical desmosomal variants.^7^^,^^8^ These episodes—commonly referred to as the inflammatory “hot phase”—are characterized by acute chest pain/troponin elevation despite angiographically normal coronary arteries, myocardial edema, and nonischemic late gadolinium enhancement (LGE) on magnetic resonance imaging (MRI).^7^

The recognition of these inflammatory flares has challenged the traditional static structural paradigm of ACM and suggests that disease progression may, in some patients, involve episodic inflammatory injury superimposed on chronic genetic susceptibility. Although the precise mechanistic basis remains incompletely defined, accumulating translational data implicate maladaptive mechanotransduction, inflammasome activation, cytokine release, and autoimmune signaling as possible mediators of this inflammatory phenotype.^9^, ^10^, ^11^ Importantly, these episodes may not merely represent epiphenomena, but may contribute directly to accelerated myocardial remodeling, scar formation, and transient amplification of ventricular arrhythmic risk.

The emerging inflammatory paradigm carries substantial diagnostic and therapeutic implications. Advances in cardiac MRI, particularly parametric tissue characterization with T2-mapping, have enabled more sensitive detection of myocardial edema and may permit recognition of active inflammatory injury beyond conventional scar imaging.^12^, ^13^, ^14^ Moreover, the identification of inflammatory signaling pathways raises the provocative hypothesis that targeted immunomodulation may represent a future disease-modifying strategy in carefully selected patients.^15^

Despite increasing recognition of myocarditis-like episodes in ACM, the concept remains inconsistently incorporated into electrophysiology practice. Patients with hot-phase presentations may be labeled as having isolated myocarditis, myocardial infarction with nonobstructive coronary arteries (MINOCA), or nonspecific myocardial injury, delaying recognition of an inherited arrhythmogenic substrate. Conversely, overinterpretation of inflammation without integration of genotype, scar burden, rhythm phenotype, and family history may lead to premature therapeutic conclusions. A focused electrophysiology-oriented synthesis is therefore needed to clarify how inflammatory activity should be interpreted within contemporary ACM diagnosis, arrhythmic risk assessment, and future therapeutic investigation.

To avoid overextension of the concept, hot-phase ACM should be regarded as a phenotype-level clue rather than a stand-alone diagnosis. The most practical implication is not immediate anti-inflammatory treatment, but a more disciplined electrophysiology evaluation that links recurrence, VA burden, genotype, scar pattern, and objective evidence of active myocardial injury.

In this review, we examine the inflammatory “hot phase” of ACM through an electrophysiology-oriented lens, integrating mechanistic, imaging, genetic, and arrhythmic evidence to propose a practical framework for diagnosis, risk assessment, and future therapeutic investigation.

We propose that the inflammatory hot phase should be conceptualized not simply as a myocarditis-like manifestation of ACM, but as a dynamic arrhythmogenic state in which transient myocardial edema, cytokine signaling, and electrical instability are superimposed on a genetically determined scar-prone substrate.

## Proposed working definition and episode boundaries

Because published definitions remain heterogeneous, we use the term hot phase as a working clinical construct rather than a formal diagnostic criterion. In this review, an active hot-phase episode refers to an acute myocarditis-like presentation in a patient with known or subsequently suspected ACM, characterized by chest pain, dyspnea, palpitations, syncope, or new arrhythmic symptoms; a rise and/or fall in cardiac troponin above the assay-specific 99th percentile; exclusion of obstructive epicardial coronary disease or another dominant ischemic mechanism when clinically appropriate; and supportive evidence of active myocardial injury such as electrocardiogram changes, cardiac magnetic resonance (CMR) edema/T2 elevation, new or expanding nonischemic LGE, focal myocardial fluorodeoxyglucose (FDG) uptake, or increased ventricular ectopy/nonsustained ventricular tachycardia (VT) (NSVT). Erythrocyte sedimentation rate (ESR) and C-reactive protein (CRP) may be elevated but are neither required nor specific, and normal inflammatory markers do not exclude ACM-related myocardial injury. Because assays and reporting vary across studies, no validated absolute troponin, ESR, or CRP threshold beyond assay-specific troponin elevation has been established for ACM hot phase. Recurrence is best defined pragmatically as 2 or more clinically discrete injury episodes separated by symptom improvement and biomarker normalization or near-normalization, rather than by a fixed interval, because available cohorts have not validated a minimum time boundary. The inter-episode period refers to clinical quiescence after resolution of acute symptoms and troponin elevation, with or without residual LGE, whereas persistent edema or ongoing biomarker elevation should be considered evidence of unresolved active injury rather than a separate episode.^7^^,^^16^, ^17^, ^18^, ^19^, ^20^, ^21^

Electrophysiology involvement is most relevant at the index or recurrent episode when there is syncope, sustained VT, NSVT, a marked increase in premature ventricular complex (PVC) burden, high-risk genotype, extensive LGE, ventricular dysfunction, family history of sudden death, or uncertainty about whether the presentation represents isolated myocarditis vs genetic ACM. This operational definition is intentionally conservative and is proposed to standardize discussion, not to replace contemporary ACM or myocarditis diagnostic criteria.^2^, ^3^, ^4^, ^5^, ^6^^,^^18^^,^^21^^,^^22^

Table 1 summarizes the proposed working definition and episode boundaries for ACM hot phase.

**Table 1:** Proposed Working Definition and Episode Boundaries for ACM Hot Phase.

Domain	Suggested Operational Criterion	Practical Caveat and Contextual References
Clinical trigger	Acute chest pain, dyspnea, palpitations, syncope, or new arrhythmic symptoms in known or suspected ACM.	Hot-phase cohorts and reviews describe myocarditis-like presentations, often with chest pain and myocardial enzyme release, whereas broader myocarditis guidance supports compatible symptoms as part of clinical suspicion.^7^^,^^16^, ^17^, ^18^
Biomarkers	Cardiac troponin above the assay-specific 99th percentile with a rise/fall pattern. ESR and CRP may be supportive.	Troponin release is central to reported hot-phase presentations; ESR/CRP are nonspecific and variably reported, so normal values should not exclude ACM-related injury.^7^^,^^16^, ^17^, ^18^
Exclusion of mimics	Exclude obstructive coronary disease or another dominant ischemic mechanism when clinically appropriate; evaluate for active infection, sarcoidosis, giant cell/eosinophilic myocarditis, and systemic inflammatory cardiomyopathy when suggested by the clinical phenotype.	ACM criteria and myocarditis guidance emphasize exclusion of alternative explanations. PET-CT and/or EMB may be appropriate when sarcoidosis, granulomatous myocarditis, giant cell myocarditis, infection, or systemic inflammatory disease is plausible.^3^^,^^18^^,^^23^^,^^24^
Imaging support	CMR edema/T2 elevation, T1/ECV abnormalities, nonischemic LGE, or focal myocardial FDG uptake.	T2 mapping supports edema/inflammation but is not ACM-specific; CMR helps characterize ACM scar patterns, while FDG-PET can identify inflammatory activity in selected ARVC/DSP cohorts and is also relevant when sarcoidosis is a mimic.^12^^,^^13^^,^^18^^,^^23^^,^^25^^,^^26^
Arrhythmic support	New or increased PVCs, NSVT, sustained VT, syncope, or device therapies during the injury window.	ACM risk assessment remains based on established markers; observational hot-phase and myocarditis-presenting ARVC data support arrhythmic vigilance, but no validated hot-phase-specific ICD threshold exists.^4^^,^^7^^,^^16^^,^^17^^,^^27^^,^^28^
Recurrence and episode boundaries	Two or more clinically discrete injury episodes separated by symptom improvement and troponin normalization or near-normalization. Persistent edema, FDG uptake, or biomarker elevation suggests unresolved active injury rather than a new episode.	This is a proposed operational boundary because published studies use heterogeneous definitions and have not validated a fixed minimum interval between hot-phase episodes.^7^^,^^16^, ^17^, ^18^^,^^25^^,^^26^

These criteria are intended for clinical communication and research standardization; they should be interpreted within genotype, family history, rhythm phenotype, imaging pattern, and exclusion of mimics.

ACM = arrhythmogenic cardiomyopathy; ARVC = arrhythmogenic right ventricular cardiomyopathy; CMR = cardiac magnetic resonance; CRP = C-reactive protein; DSP = desmoplakin; ECV = extracellular volume; EMB = endomyocardial biopsy; ESR = erythrocyte sedimentation rate; FDG = fluorodeoxyglucose; ICD = implantable cardioverter-defibrillator; LGE = late gadolinium enhancement; NSVT = nonsustained ventricular tachycardia; PET-CT = positron emission tomography–computed tomography; PVC = premature ventricular complex; VT = ventricular tachycardia.

## Molecular mechanisms: from desmosomal dysfunction to inflammatory activation

Emerging evidence suggests that the inflammatory phenotype observed during the “hot phase” of ACM may arise from a complex interplay between structural desmosomal dysfunction, maladaptive mechanotransduction, innate immune activation, and autoimmune signaling. Although the precise temporal and causal relationships among these processes remain incompletely elucidated, available translational data support a multifactorial mechanistic framework linking genetic substrate to inflammatory myocardial injury.

## Desmosomal instability and aberrant mechanotransduction

The molecular basis of ACM is most commonly rooted in pathogenic variants affecting desmosomal proteins, including plakoglobin, DSP, plakophilin-2 (PKP2), and desmoglein-2 (DSG2), among others.^29^, ^30^, ^31^, ^32^, ^33^, ^34^ These proteins maintain structural integrity at the intercalated disc, permitting cardiomyocytes to tolerate repetitive mechanical stress during contraction.^29^^,^^30^ Disruption of this architecture impairs intercellular mechanical coupling and increases susceptibility to stress-induced microinjury.

Beyond structural destabilization, desmosomal disruption appears to alter intracellular signaling pathways central to myocardial homeostasis. Experimental studies have demonstrated that impaired desmosomal integrity may promote nuclear translocation of plakoglobin, resulting in suppression of canonical Wnt/β-catenin signaling.^35^^,^^36^ This signaling shift has been associated with enhanced adipogenic and fibrogenic transcriptional programming, providing 1 mechanistic explanation for the characteristic fibro-fatty myocardial replacement observed in ACM.^36^^,^^37^ However, the extent to which this pathway independently drives disease progression in human phenotypes remains incompletely established.

## Inflammasome activation and cytokine signaling

In addition to chronic structural remodeling, recent preclinical investigations suggest that desmosomal injury may trigger innate immune activation through inflammasome-mediated pathways. Mechanical stress and repetitive cardiomyocyte microinjury may promote release of intracellular danger-associated molecular patterns, thereby activating pattern-recognition receptors and downstream inflammasome complexes, including the NLRP3 inflammasome.^9^^,^^10^^,^^20^ Activated inflammasome signaling promotes cleavage and secretion of proinflammatory cytokines, most notably interleukin (IL)-1β and IL-18, which may amplify local inflammatory recruitment and myocardial injury.

Importantly, evidence supporting inflammasome activation in ACM remains largely derived from experimental and translational models, and whether inflammasome signaling serves as a primary pathogenic driver vs secondary response to cardiomyocyte injury remains a matter of active debate. Nevertheless, these observations support the hypothesis that inflammatory signaling may contribute meaningfully to disease propagation in selected inflammatory-prone ACM phenotypes.

This distinction is clinically important because mechanistic plausibility alone should not be interpreted as evidence that inflammasome-directed therapy is indicated in routine ACM care. At present, these pathways are best viewed as hypothesis-generating targets that require genotype-stratified validation and correlation with arrhythmic outcomes.

## Autoimmune and humoral contributions

Additional mechanistic complexity may arise from adaptive immune activation. Prior investigations have identified circulating anti-DSG2 autoantibodies in patients with ACM, suggesting that progressive desmosomal disruption may expose normally sequestered intracellular or junctional antigens, thereby initiating autoimmune sensitization.^11^ These antibodies have been associated with electrical instability and arrhythmic burden in observational studies and may contribute to impaired gap junction function and connexin remodeling.^11^

However, the pathogenic significance of anti-DSG2 antibodies remains incompletely understood. Whether these autoantibodies serve as true mediators of disease progression, epiphenomena of ongoing myocardial injury, or biomarkers of disease severity has yet to be definitively determined.

The anti-DSG2 signal has been reported across complementary human and animal data sets, but the interpretation has evolved. In the original study, anti-DSG2 antibodies were identified in 12 of 12 definite arrhythmogenic RV cardiomyopathy (ARVC) patients in a primary cohort, 25 of 25 definite ARVC patients in a validation cohort, 7 of 8 borderline ARVC subjects, and 10 of 10 Boxer dogs with ARVC; antibody levels correlated with PVC burden (r = 0.70), and purified antibodies impaired gap-junction function in vitro.^11^ A subsequent multicenter study that included 77 ARVC patients, 91 myocarditis/DCM patients, 27 systemic immune-mediated disease patients, and 50 controls found anti-DSG2 positivity in 51% of ARVC patients and 48% of myocarditis/DCM patients, suggesting that anti-DSG2 may reflect immune reactivity to desmosomal constituents but is not disease-specific enough for stand-alone diagnosis.^38^ Mechanistically, desmosomal injury may expose cryptic DSG2 epitopes, promote antigen presentation, and generate adaptive immune responses that secondarily worsen intercalated-disc and connexin-43 remodeling^11^^,^^39^^,^^40^; however, whether these antibodies are primary mediators, amplifiers, or biomarkers of myocardial injury remains unresolved.^11^^,^^38^^,^^41^

## Toward an integrated inflammatory model

**Figure 1:**
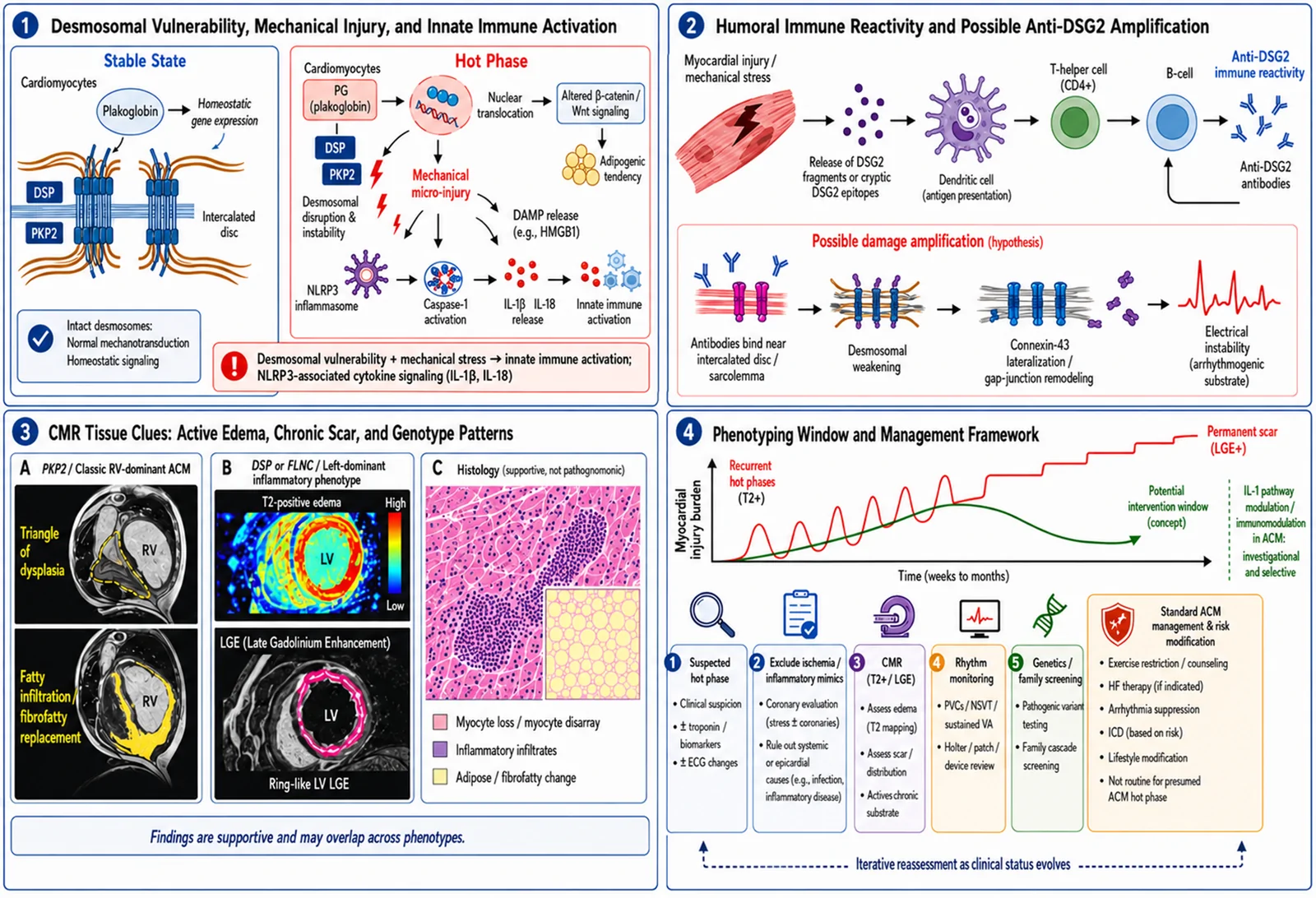
This 4-Panel Figure Integrates Mechanistic and Clinical Aspects of the ACM Hot-Phase Paradigm Panel 1 illustrates how desmosomal vulnerability and mechanical micro-injury may promote maladaptive plakoglobin/Wnt signaling, adipogenic tendency, DAMP release, NLRP3 inflammasome activation, caspase-1 activation, and IL-1β/IL-18 release. Panel 2 depicts potential humoral immune amplification through anti-DSG2 immune reactivity, with desmosomal weakening, connexin-43 remodeling, and electrical instability shown as hypothesis-generating downstream consequences. Panel 3 highlights imaging and histologic clues across phenotypes, including classic RV-dominant disease, left-dominant inflammatory-prone patterns with T2-positive edema and ring-like LV LGE, and supportive—but not pathognomonic—histology. Panel 4 summarizes a practical phenotyping and management framework: suspect hot phase clinically; exclude ischemic and inflammatory mimics; use CMR to characterize active edema vs chronic scar; intensify rhythm monitoring; pursue genetics and family screening; and apply standard ACM management and risk modification, while recognizing that immunomodulation remains investigational and selective. ACM = arrhythmogenic cardiomyopathy; CMR = cardiac magnetic resonance; DAMP = danger-associated molecular pattern; DSG2 = desmoglein-2; DSP = desmoplakin; ECG = electrocardiography; HF = heart failure; ICD = implantable cardioverter-defibrillator; IL = interleukin; LGE = late gadolinium enhancement; LV = left ventricle; NSVT = nonsustained ventricular tachycardia; PKP2 = plakophilin-2; PVC = premature ventricular complex; RV= right ventricle; FLNC = filamin C; VA = ventricular arrhythmia.

Taken together, current mechanistic data support a conceptual model in which genetically mediated desmosomal instability predisposes to mechanical injury, maladaptive intracellular signaling, and inflammatory amplification. In susceptible patients, episodic inflammatory activation may transiently exacerbate myocardial injury and electrical instability, thereby accelerating progression toward fibrosis, scar formation, and ventricular arrhythmogenesis. Although substantial mechanistic uncertainty persists, this integrated framework provides biologic plausibility for the emerging concept of ACM as a disease characterized not solely by structural degeneration, but also by dynamic inflammatory activation. This integrated mechanistic, imaging, and clinical framework is summarized in Figure 1.

## Advanced imaging: visualizing the inflammatory phenotype

Recognition of the inflammatory “hot phase” in ACM has been facilitated largely by advances in CMR, which permit increasingly refined characterization of myocardial tissue composition beyond conventional structural assessment. Whereas traditional imaging approaches emphasize chamber size, systolic function, and scar burden, contemporary parametric mapping techniques provide insight into dynamic myocardial processes including edema and active inflammation.

## Late gadolinium enhancement and chronic structural substrate

LGE remains the cornerstone of tissue characterization in ACM and identifies regions of replacement fibrosis and established myocardial scar. In affected patients, LGE frequently demonstrates subepicardial or mid-myocardial distribution in a nonischemic pattern, reflecting chronic fibro-fatty remodeling and replacement fibrosis.^2^^,^^3^^,^^5^^,^^13^^,^^42^ However, although LGE effectively characterizes chronic scar burden, it cannot reliably distinguish active inflammatory injury from chronic residual fibrosis.

## T2 mapping and detection of active myocardial edema

T2-based parametric mapping has emerged as a promising adjunctive modality for detection of active myocardial edema and inflammatory injury. Elevated myocardial T2 relaxation times reflect increased free water content and may identify transient inflammatory myocardial injury during acute myocarditis-like presentations.^12^, ^13^, ^14^^,^^42^^,^^43^ In patients with suspected inflammatory ACM phenotypes, particularly those presenting with chest pain and troponin elevation, T2 abnormalities may support the presence of active inflammatory myocardial involvement beyond chronic scar visualization.

Nevertheless, important limitations warrant consideration. Elevated T2 values are not specific to ACM and may be observed across numerous inflammatory and infiltrative myocardial disease states, including viral myocarditis, sarcoidosis, and systemic inflammatory cardiomyopathies. Accordingly, T2 mapping should be interpreted within the broader clinical, genetic, and phenotypic context^18^, ^19^, ^20^, ^21^^,^^42^^,^^43^ rather than as a stand-alone discriminator of ACM-related inflammation.

## Viral immune response, FDG-positron emission tomography/computed tomography, and endomyocardial biopsy

Earlier ARVC/ACM literature considered viral infection as a potential inflammatory trigger or contributor. Cardiotropic viral genomes were detected in some historical series, whereas other studies did not detect enteroviral genome, leaving viral involvement as a possible trigger, mimic, or bystander rather than an established unifying mechanism.^44^^,^^45^ This distinction remains clinically relevant because a patient with a pathogenic ACM variant may still develop true viral myocarditis, and conversely recurrent myocarditis-like injury may reveal an inherited desmosomal substrate. Therefore, viral testing and endomyocardial biopsy (EMB)-based viral polymerase chain reaction should be individualized rather than assumed from phenotype alone.^18^

FDG-positron emission tomography/computed tomography (PET/CT) is not required for routine ACM diagnosis, but it is useful when the differential includes cardiac sarcoidosis, extracardiac inflammatory disease, active myocarditis, or when CMR findings and clinical features are discordant. In cardiac sarcoidosis, CMR and FDG-PET provide complementary information: CMR characterizes scar and tissue injury, whereas FDG-PET can identify metabolically active inflammation and extracardiac disease; a meta-analysis of 33 studies reported cardiac MRI sensitivity/specificity of 95%/85% and FDG-PET sensitivity/specificity of 84%/82% for cardiac sarcoidosis.^23^ Limited ACM-specific PET data suggest that myocardial FDG uptake can be present in ARVC/ACM, but interpretation is challenging because physiologic uptake and sarcoidosis/myocarditis mimics may confound results.^25^ A prospective DSP cardiomyopathy study of 10 DSP and 4 titin participants found no significant differences in white blood cell count, ESR, high-sensitivity CRP, high-sensitivity troponin, or myocardial FDG uptake in clinically ambulatory patients, whereas proteomic analyses suggested enrichment of inflammatory and pyroptosis pathways; importantly, the authors emphasized that future work should evaluate patients during myocarditis-like episodes rather than quiescent periods.^26^

EMB should be discussed explicitly but selectively. EMB is not required to diagnose most ACM cases and may be limited by sampling error, particularly in patchy left ventricular (LV) or subepicardial disease. However, EMB becomes important when the presentation is fulminant, when there is new high-grade conduction disease, rapidly progressive heart failure, refractory VAs, suspected giant cell myocarditis, eosinophilic myocarditis, sarcoidosis, infectious myocarditis, or when immunosuppression is being considered.^18^, ^19^, ^20^, ^21^ Extensive LGE with myocarditis-like injury is not specific for ACM; giant cell myocarditis, sarcoidosis, eosinophilic myocarditis, immune-checkpoint-inhibitor myocarditis, and systemic inflammatory cardiomyopathies must remain in the differential.^18^

This approach is consistent with contemporary myocarditis guidance, in which CMR is interpreted as 1 component of a broader diagnostic pathway rather than a substitute for clinical context, coronary evaluation, rhythm assessment, and selective follow-up imaging.^18^, ^19^, ^20^, ^21^^,^^42^ In recurrent troponin-positive presentations, the presence of nonischemic LGE plus an ACM-compatible family, genetic, or arrhythmic phenotype should lower the threshold for inherited cardiomyopathy evaluation.

## Genotype-specific imaging patterns

Distinct imaging phenotypes may further support recognition of inflammatory-prone ACM subtypes. DSP-associated cardiomyopathy, in particular, has been linked to extensive LV fibrosis with characteristic circumferential or “ring-like” subepicardial LGE patterns, often associated with recurrent myocarditis-like presentations and heightened arrhythmic risk.^8^^,^^27^^,^^46^, ^47^, ^48^, ^49^ These imaging signatures may reflect genotype-specific disease biology and support the concept that inflammatory manifestations may cluster within particular molecular subgroups.

Genotype-specific interpretation is particularly important because inflammatory activity appears to cluster in selected molecular subtypes rather than representing a uniform feature of all ACM. DSP cardiomyopathy is increasingly recognized as a distinct fibrotic-inflammatory phenotype characterized by recurrent myocarditis-like episodes, left-dominant involvement, subepicardial or ring-like LV fibrosis, and substantial VA burden.^8^^,^^27^^,^^31^^,^^46^, ^47^, ^48^, ^49^, ^50^ filamin C (FLNC)- and desmin (DES)-associated phenotypes may also show left-dominant arrhythmogenic remodeling, whereas classic PKP2-associated disease more often follows a right-dominant or biventricular pattern.^27^^,^^34^^,^^50^^,^^51^ Accordingly, recurrent troponin-positive chest pain with nonischemic LV LGE should prompt consideration of genetic ACM, particularly DSP cardiomyopathy, even when conventional RV diagnostic criteria are not fully met.^2^^,^^3^^,^^5^^,^^6^^,^^46^^,^^47^

Importantly, the hot-phase literature is enriched for DSP cardiomyopathy, but the inflammatory phenotype should not be reduced to DSP myocarditis alone. In the systematic review of 103 reported ACM hot-phase patients, DSP was the most common disease gene (69%), followed by PKP2 (9%) and DSG2 (6%); LV epicardial LGE was reported in 86% of cases, reinforcing the left-dominant DSP signal while also demonstrating genotype heterogeneity.^7^^,^^17^ In a separate registry series, 12 women initially diagnosed with myocarditis were later diagnosed with ARVC; all had troponin elevation, 42% had reduced LV function, and DSP variants were frequent, again supporting the myocarditis-like entry point into genetic ACM.^28^

PKP2-associated ACM, in contrast, often presents earlier with right-dominant or biventricular arrhythmogenic disease, and exercise-related mechanical stress has stronger biologic and clinical plausibility in classic desmosomal ARVC than in many DSP presentations.^4^^,^^32^^,^^50^^,^^52^, ^53^, ^54^, ^55^ Myocarditis-like injury has nevertheless been described in early or atypical PKP2 presentations, so the absence of a DSP variant should not exclude the diagnosis when recurrent troponin-positive injury, VAs, and compatible imaging or family history are present. DSG2-associated disease may present with biventricular involvement and, in rare homozygous or severe variants, prominent inflammatory features; FLNC-associated ACM more often demonstrates left-dominant scar and VA risk, but the frequency and biology of true hot-phase episodes remain less well defined than for DSP.^16^^,^^17^^,^^27^^,^^33^^,^^34^^,^^39^^,^^50^^,^^51^

## Imaging as a translational biomarker

Beyond diagnosis, advanced imaging may ultimately serve as a translational biomarker for disease activity and therapeutic monitoring. If inflammatory pathways prove mechanistically relevant to ACM progression, serial CMR with parametric mapping may offer a noninvasive method for tracking inflammatory burden and evaluating response to future targeted immunomodulatory interventions. However, prospective validation of imaging-guided therapeutic strategies remains necessary before incorporation into routine clinical management paradigms.

## The hot phase as a dynamic electrophysiologic substrate

For electrophysiologists, the central significance of the ACM hot phase is that arrhythmic risk may fluctuate over time rather than simply accumulate in parallel with scar burden. During inflammatory flares, myocardial edema, cytokine-mediated ion-channel remodeling, calcium-handling abnormalities, and connexin redistribution may transiently increase electrical instability on top of an inherited arrhythmogenic substrate. This creates a dynamic electrophysiologic state in which ventricular ectopy, NSVT, sustained VAs, or syncope may cluster during periods of active myocardial injury, even when ventricular dysfunction or LGE burden appears modest.

Although the inflammatory “hot phase” is often recognized clinically through chest pain, troponin elevation, and CMR evidence of edema, its most important implication for electrophysiologists is the possibility of transient arrhythmic vulnerability superimposed on a fixed genetic substrate.^4^^,^^7^^,^^16^^,^^27^ Inflammation may amplify arrhythmogenesis through several convergent mechanisms, including cytokine-mediated ion-channel remodeling, altered calcium handling, connexin-43 redistribution, conduction slowing, and spatial heterogeneity between edematous but viable myocardium and established scar.^9^^,^^11^^,^^20^^,^^27^^,^^39^^,^^40^ This creates a dynamic substrate in which ventricular ectopy, NSVT, or sustained VAs may cluster during periods of active myocardial injury rather than reflecting scar burden alone.^4^^,^^27^^,^^47^^,^^48^^,^^56^, ^57^, ^58^ Recognition of this temporal dissociation is clinically important because a patient with modest ventricular dysfunction or limited LGE may still experience heightened arrhythmic risk during an inflammatory flare.^4^^,^^46^, ^47^, ^48^^,^^56^, ^57^, ^58^

## Magnitude of arrhythmic risk and implantable cardioverter-defibrillator decision-making

Available evidence supports the concept of heightened arrhythmic vulnerability during inflammatory injury, but it does not yet provide a validated hot-phase-specific HR or implantable cardioverter-defibrillator (ICD) threshold. In the study by Bariani et al^17^, no sustained VA or sudden death occurred during the acute hot-phase episode itself, despite substantial downstream diagnostic reclassification and frequent DSP-associated disease; this argues against treating every troponin-positive episode as an automatic ICD indication. Conversely, DSP cardiomyopathy cohorts and reviews associate myocarditis-like episodes, LV scar, and VAs with adverse outcomes, suggesting that hot phases may identify a high-risk phenotype over time rather than define an isolated acute event risk by themselves.^16^^,^^27^^,^^46^^,^^47^

Accordingly, intensified rhythm surveillance during and after an active episode should be framed as a risk-clarification strategy, not as a proven intervention that independently improves outcomes. Practical consequences include capturing transient NSVT/PVC escalation, documenting sustained VT or syncope during active injury, reassessing arrhythmia burden after biomarker and edema resolution, and avoiding premature reassurance when ventricular function is preserved but genotype and scar burden are high risk. ICD implantation should remain anchored to established ACM risk markers and secondary prevention indications. Active inflammation may influence timing, shared decision-making, temporary protection strategies in selected high-risk patients, and the urgency of reassessment, but current data do not justify lowering ICD thresholds solely because edema or troponin elevation is present.^4^^,^^16^^,^^18^^,^^27^^,^^47^

The hot-phase concept also reframes rhythm monitoring in ACM. Ambulatory electrocardiographic surveillance during and shortly after troponin-positive inflammatory episodes may provide information that is not captured by routine interval monitoring in clinically quiescent periods. In this setting, increasing PVC burden, polymorphic ectopy, new NSVT, syncope, or sustained VA should be interpreted in the context of genotype, LGE burden, ventricular function, and family history rather than as isolated electrical findings.^4^^,^^16^^,^^27^^,^^47^

A pragmatic approach is to document the inflammatory window with serial troponin or inflammatory markers when clinically appropriate, obtain ambulatory monitoring after symptom stabilization, and compare arrhythmic burden with prior quiescent periods whenever available. This framing may help distinguish transient inflammatory amplification from progression of the fixed scar-mediated substrate, while avoiding the unsupported assumption that inflammation alone determines ICD candidacy.

## Clinical electrophysiology implications

The inflammatory hot-phase paradigm has several practical implications for electrophysiology care. First, rhythm monitoring should be intensified during and shortly after troponin-positive inflammatory episodes, because arrhythmic burden during these windows may not be captured by routine surveillance performed during clinically quiescent periods. Second, VAs occurring during a hot phase should not be dismissed as isolated myocarditis-related ectopy, particularly when accompanied by recurrent injury, nonischemic LV LGE, family history, or a pathogenic ACM-associated variant. Third, ICD decisions should continue to follow established ACM risk markers, but active inflammation may provide complementary temporal information by identifying periods of transiently amplified risk. Finally, exercise restriction during active inflammatory injury is biologically plausible and should be integrated with biomarker normalization, rhythm reassessment, and follow-up imaging before return to higher-intensity activity.

## Clinical recognition and translational implications

Recognition of inflammatory activation in ACM has important implications for the evolving clinical management of this disease. Traditionally, ACM has been approached primarily through structural and arrhythmic risk stratification, with therapeutic strategies focused on sudden death prevention and management of progressive ventricular dysfunction.^4^ However, increasing recognition of transient inflammatory exacerbations suggests that selected patients may experience dynamic periods of heightened biologic activity that are not fully captured by conventional static risk models.

From a diagnostic standpoint, awareness of the inflammatory “hot phase” may improve recognition of ACM in patients presenting with myocarditis-like syndromes, particularly when recurrent chest pain, troponin elevation, and nonischemic CMR abnormalities occur in the absence of obstructive coronary disease, a constellation increasingly recognized in inflammatory-prone ACM phenotypes.^7^^,^^13^ In these contexts, integration of imaging phenotype, family history, and genetic testing may facilitate earlier diagnosis of inflammatory-prone ACM subtypes.

Because the hot phase frequently mimics acute myocarditis, cardiac sarcoidosis, MINOCA, and other inflammatory cardiomyopathies, a structured differential diagnostic approach is essential to avoid both under-recognition of genetic ACM and inappropriate attribution of recurrent injury to isolated myocarditis.^3^^,^^7^^,^^16^^,^^17^^,^^38^^,^^46^

Table 2 provides representative human data informing the inflammatory ACM hot-phase phenotype, emphasizing that most evidence remains observational and genotype-enriched rather than definitive for management thresholds. Table 3 summarizes practical clinical clues that help distinguish ACM hot phase from common mimics, including viral myocarditis, cardiac sarcoidosis, and MINOCA/ischemia.

**Table 2:** Representative Human Data Informing the Inflammatory ACM Hot-Phase Phenotype

Study/Source (Reference)	Patients/Design	Key Inflammatory/Arrhythmic Findings	Management Implication
Bariani et al systematic review^7^	Systematic review of 9 studies including 103 ACM patients with hot-phase episodes	Mean age 26 ± 14 years; LV epicardial LGE in 86%; DSP 69%, PKP2 9%, DSG2 6%; role in disease progression and arrhythmic risk remains unclear.	Defines phenotype/genotype enrichment, but not hot-phase-specific ICD thresholds.
Bariani et al clinical series^17^	Single-center series of 23 ACM patients with chest pain and/or myocardial enzyme release with normal coronary arteries, identified among 560 ACM probands/family members	Mean age at first episode 27 ± 16 years; CMR during the acute phase showed edema/hyperemia in 58% and LGE in 92%; no sustained VA or sudden death during hot-phase episodes; 12 additional patients achieved an ACM diagnosis during follow-up.	Supports ECG/rhythm surveillance, longitudinal phenotyping, genetic/family evaluation, and reclassification rather than automatic acute ICD escalation based only on an acute hot phase.
Scheel et al^28^	Johns Hopkins ARVC Registry: 12 women initially diagnosed with myocarditis and later ARVC	All had troponin elevation; chest pain in 58%; ventricular arrhythmia in 25%; reduced LV function in 42%; pathogenic variants in 92% (10 DSP, 1 DSG2).	Genetic ACM, especially DSP-related disease, should be considered after myocarditis-like presentations with LV involvement.
Protonotarios et al^25^	16 definite ARVC patients undergoing FDG-PET	Positive myocardial FDG uptake in 7/16 (44%); 2 had cardiac sarcoidosis on biopsy, underscoring limited specificity.	FDG-PET may identify inflammatory activity but is not ACM-specific; abnormal uptake should prompt evaluation for sarcoidosis and other mimics.
Divakaran et al^26^	Prospective ambulatory cohort: 10 DSP vs 4 TTN cardiomyopathy patients with FDG-PET/CT and inflammatory biomarkers	No significant group differences in WBC count, ESR, hsCRP, hs-troponin, or FDG-PET findings; 3 DSP and 2 TTN participants had nonspecific FDG uptake; inflammatory/pyroptosis pathways were enriched by proteomic/miRNA analyses.	Quiescent ambulatory testing may miss episode-specific inflammation; future studies should sample during myocarditis-like episodes.

DSG2 = desmoglein-2; ECG = electrocardiogram; hsCRP = high-sensitivity C-reactive protein; LV = left ventricular; miRNA = microRNA; PKP2 = plakophilin-2; TTN = titin; VA = ventricular arrhythmia; WBC = white blood cell count; other abbreviations as in Table 1.

**Table 3:** Clinical Clues Distinguishing Arrhythmogenic Cardiomyopathy Hot Phase From Common Mimics

Feature	ACM Hot Phase	Viral Myocarditis	Cardiac Sarcoidosis	MINOCA/Ischemia
Typical clue	Recurrent injury, family history, VA, pathogenic ACM variant	Viral prodrome or isolated myocarditis syndrome; recurrence can occur but usually lacks ACM genotype/family-history clues	Systemic sarcoidosis, high-grade AV block, basal septal or multifocal disease	Ischemic symptoms or vascular risk factors
Coronary evaluation	Usually normal after ischemia is excluded	Usually normal	Usually normal unless concomitant CAD or another ischemic mechanism is present	Plaque disruption, spasm, embolism, or microvascular dysfunction may be present
CMR edema	T2 elevation during active flare	T2 elevation during acute myocardial inflammation	Patchy active inflammation possible	Edema follows ischemic territory when infarction is present
LGE pattern	Subepicardial/mid-wall; ring-like LV LGE suggests DSP	Often subepicardial or mid-wall, commonly inferolateral	Patchy basal septal/lateral or multifocal	Subendocardial/transmural coronary distribution
Genetic/family clues	DSP, PKP2, DSG2, FLNC, DES; family history of ACM/SCD	Usually absent; familial myocarditis is uncommon	Usually absent for ACM/SCD pattern; systemic sarcoidosis history may be present	Usually absent
Arrhythmic profile	PVCs, NSVT, sustained VT; risk may be disproportionate to EF	Variable ventricular ectopy; higher-risk arrhythmias possible in severe or fulminant myocarditis	AV block, VT, ventricular dysfunction, and multifocal scar-related arrhythmias	Ischemia-related ventricular arrhythmias

Features are representative clinical patterns and should be interpreted collectively rather than as diagnostic criteria. ACM hot-phase clues are synthesized from contemporary ACM diagnostic and management frameworks and hot-phase cohorts/reviews.^3^^,^^4^^,^^7^^,^^16^^,^^17^^,^^46^ Viral myocarditis features are supported by contemporary myocarditis guidance and CMR myocardial inflammation criteria.^18^^,^^42^ Cardiac sarcoidosis features are supported by cardiac sarcoidosis imaging and diagnostic guidance.^23^^,^^59^ MINOCA/ischemia features are supported by the AHA scientific statement on myocardial infarction with nonobstructive coronary arteries.^60^

AV = atrioventricular; CAD = coronary artery disease; DES = desmin; EF = ejection fraction; FLNC = filamin C; MINOCA = myocardial infarction with nonobstructive coronary arteries; PVC = premature ventricular complex; SCD = sudden cardiac death; other abbreviations as in Tables 1 and 2.

From a translational perspective, the emerging inflammatory paradigm raises the possibility that future risk stratification frameworks may incorporate dynamic biomarkers of disease activity in addition to conventional structural and electrophysiologic parameters. Whether inflammatory biomarkers, circulating autoantibodies, or serial imaging metrics can meaningfully improve prediction of arrhythmic or structural progression remains an area of active investigation.

Importantly, the hot-phase concept should not be interpreted as replacing established ACM risk stratification frameworks. Decisions regarding ICD implantation should continue to be guided by conventional risk markers, including prior sustained VAs, unexplained syncope, ventricular dysfunction, genotype, family history, burden of ventricular ectopy, and extent of myocardial scar.^4^^,^^27^^,^^47^ However, inflammatory activity may provide complementary temporal information by identifying periods during which arrhythmic risk may be transiently amplified. Future studies should determine whether edema-positive inflammatory flares independently predict VAs after adjustment for LGE burden, ventricular function, and genotype.^16^^,^^27^^,^^47^

Ultimately, incorporation of inflammatory phenotyping into ACM evaluation may support a broader transition toward precision-based disease characterization integrating genotype, imaging phenotype, and biologic activity into individualized management strategies.

Clinically, a suspected ACM hot phase should trigger a stepwise evaluation rather than isolated treatment as nonspecific myocarditis. First, ischemia should be excluded when clinically appropriate. Second, CMR should assess both active injury and chronic substrate using T2 mapping, T1 mapping/extracellular volume when available, and LGE distribution. Third, rhythm assessment with ambulatory monitoring should be performed to identify ventricular ectopy, NSVT, or sustained VAs during the inflammatory window. Fourth, genetic testing and cascade family evaluation should be considered when imaging pattern, recurrence, family history, or arrhythmic features suggest inherited cardiomyopathy. Finally, management should integrate conventional ACM risk markers with dynamic evidence of active inflammation, while recognizing that anti-inflammatory treatment remains investigational outside selected clinical contexts.^3^^,^^4^^,^^16^^,^^17^^,^^27^^,^^38^

A simplified electrophysiology-oriented take-home framework for suspected ACM hot phase is summarized in the Central Illustration, integrating the predisposing substrate, dynamic inflammatory phase, downstream progression, and practical clinical implications. The more detailed mechanistic, imaging, and management synthesis is provided in Figure 1.

**Central Illustration:**
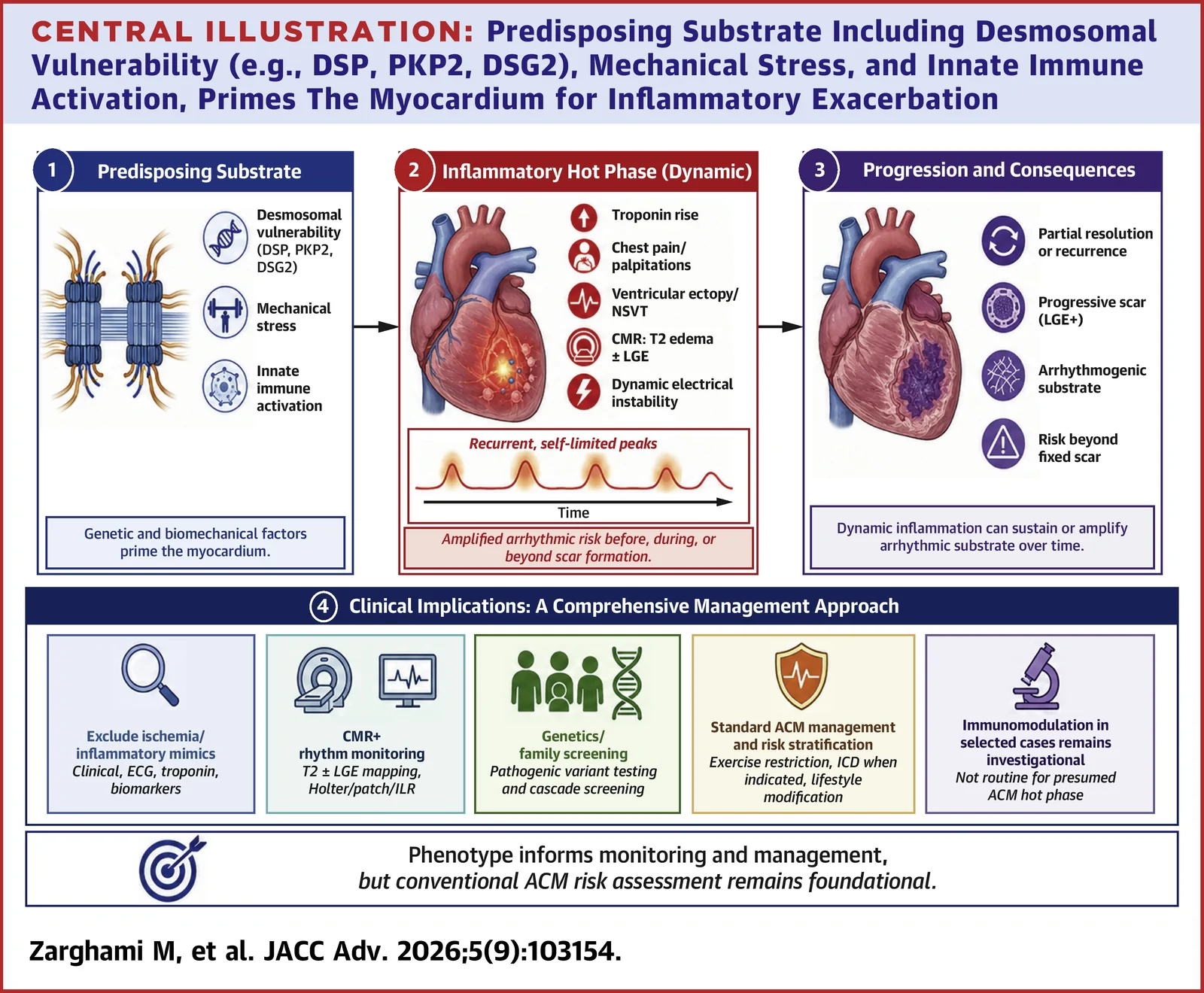
Predisposing Substrate Including Desmosomal Vulnerability (e.g., DSP, PKP2, DSG2), Mechanical Stress, and Innate Immune Activation, Primes The Myocardium for Inflammatory Exacerbation During the inflammatory hot phase, patients may develop chest pain or palpitations, troponin elevation, ventricular ectopy or NSVT, CMR evidence of T2 edema with or without LGE, and dynamic electrical instability. Over time, episodes may partially resolve or recur, while repeated injury may promote progressive scar, a persistent arrhythmogenic substrate, and risk beyond fixed scar alone. The management layer emphasizes exclusion of ischemic or inflammatory mimics, CMR plus rhythm monitoring, genetics and family screening, standard ACM management and conventional risk stratification, and the continued investigational status of immunomodulation for presumed ACM hot phase. ACM = arrhythmogenic cardiomyopathy; CMR = cardiac magnetic resonance; DSG2 = desmoglein-2; DSP = desmoplakin; ECG = electrocardiography; ICD = implantable cardioverter-defibrillator; ILR = implantable loop recorder; LGE = late gadolinium enhancement; NSVT = nonsustained ventricular tachycardia; PKP2 = plakophilin-2.

## Therapeutic implications: toward targeted immunomodulation

The recognition of inflammatory activation in selected ACM phenotypes raises important questions regarding whether inflammatory signaling may represent a therapeutically modifiable component of disease progression. Although current ACM management remains centered on arrhythmic risk stratification, heart failure therapy, lifestyle modification, and ICD placement, these strategies are largely reactive and do not directly address the molecular mechanisms underlying disease progression.^4^

Lifestyle modification remains a critical disease-modifying intervention in ACM and should be explicitly integrated into the inflammatory hot-phase framework. Competitive or high-intensity endurance exercise may increase mechanical stress on an already vulnerable desmosomal substrate and has been associated with earlier disease expression, greater arrhythmic penetrance, and adverse VA outcomes in ACM.^4^^,^^52^^,^^53^ During suspected hot-phase episodes, temporary restriction from intense exercise is biologically plausible and clinically prudent, with return-to-activity decisions guided by symptom resolution, biomarker normalization, rhythm monitoring, and follow-up imaging when appropriate.^4^^,^^52^

This recommendation should be interpreted carefully. Exercise restriction is not proposed only during hot phases; avoidance of competitive or high-intensity endurance exercise is already part of established ACM management, particularly in classic desmosomal/PKP2-associated ARVC where exercise-related penetrance and arrhythmic outcomes are most compelling.^4^^,^^52^^,^^53^ During an active hot phase, temporary abstention from moderate-to high-intensity exercise is a myocarditis-informed precaution until symptoms improve, troponin normalizes, arrhythmic burden is reassessed, and active edema resolves or clearly improves. Contemporary myocarditis guidance provides a useful clinical analogy: patients with clinically overt myocarditis are generally advised to avoid strenuous physical activity for 3 to 6 months and to undergo reassessment with ventricular imaging, ambulatory electrocardiographic monitoring, and exercise testing before resuming strenuous exercise.^18^ These restrictions are based largely on expert consensus and have not been validated specifically for ACM hot phases; therefore, they should inform, rather than rigidly determine, individualized return-to-activity decisions. This should not be read as a uniform gene-specific prohibition for all ACM subtypes, or as evidence that DSP hot phases are usually exercise-triggered; rather, it is a conservative safety recommendation individualized by genotype, phenotype, prior arrhythmias, athletic exposure, and recovery trajectory.^4^^,^^18^^,^^52^^,^^53^

## Rationale for immunomodulatory intervention

Given emerging evidence implicating innate immune activation, inflammasome signaling, and IL-1–mediated cytokine cascades in inflammatory-prone ACM phenotypes, targeted immunomodulation has been proposed as a potential future disease-modifying strategy.^9^^,^^10^^,^^15^ In particular, IL-1 blockade has gained increasing interest across inflammatory cardiovascular disease states because of its central role in amplifying myocardial inflammatory injury.

Preliminary translational and clinical evidence from myocarditis and systemic inflammatory cardiomyopathies suggests that IL-1 inhibition may attenuate inflammatory myocardial injury in selected contexts.^15^ However, direct evidence supporting its efficacy in ACM remains absent, and extrapolation to genetically mediated inflammatory cardiomyopathy should therefore be approached cautiously.

The therapeutic discussion should therefore be broadened beyond IL-1. IL-1 blockade is highlighted because inflammasome and IL-1 biology provide a plausible mechanistic bridge from cardiomyocyte injury to cytokine-mediated electrical instability, and because early human myocarditis experience has made IL-1 signaling a testable pathway.^15^ However, ACM-specific evidence remains absent. Corticosteroids and broader immunosuppression have a clearer role in specific inflammatory cardiomyopathies such as giant cell myocarditis, eosinophilic myocarditis, cardiac sarcoidosis, immune-checkpoint-inhibitor myocarditis, or biopsy-proven immune-mediated myocarditis after exclusion of active viral replication, but they cannot be recommended routinely for presumed ACM hot phases without tissue diagnosis or highly selected expert evaluation.^18^ ESR and CRP may help document systemic inflammation and monitor response in selected cases, but they are nonspecific, inconsistently elevated, and insufficient to guide immunomodulatory therapy in isolation.^18^^,^^26^

Animal and translational ACM studies support the biologic plausibility of immune modulation, including innate immune activation, cytokine signaling, and desmosome/intercalated-disc remodeling, but human evidence is limited to observational cohorts, case reports, small series, and extrapolation from myocarditis or sarcoidosis. Thus, any discussion of steroids, IL-1 blockade, or other immunomodulators should be presented as hypothesis-generating and ideally restricted to multidisciplinary cardiomyopathy/myocarditis programs or prospective protocols.^9^^,^^10^^,^^15^^,^^16^^,^^18^^,^^27^

In preclinical ACM models, immune-directed interventions have been tested more directly than in humans. In DSG2-mutant murine models and complementary in vitro systems, NF-κB activation in cardiomyocytes has been linked to inflammatory cytokine production, CCR2+ macrophage recruitment, myocardial injury, fibrosis, contractile dysfunction, electrocardiogram abnormalities, and arrhythmic features; pharmacologic or genetic inhibition of NF-κB signaling and disruption of CCR2+ macrophage recruitment attenuated several of these phenotypic manifestations. These animal data support biologic plausibility for immune-targeted therapy, but they remain model-dependent and should not be interpreted as evidence that corticosteroids, IL-1 blockade, NF-κB inhibition, or macrophage-directed therapies are clinically indicated for routine ACM hot-phase care.^9^^,^^61^

## Theoretical therapeutic window

One conceptual implication of the inflammatory “hot phase” paradigm is the possibility that transient periods of active myocardial edema may represent a biologically relevant therapeutic window during which inflammatory intervention could theoretically attenuate downstream fibro-fatty remodeling. Advanced tissue characterization with T2-mapping may, in the future, facilitate identification of this putative inflammatory phase and allow temporally targeted intervention.

At present, however, no prospective studies have established whether imaging-guided immunomodulation alters arrhythmic burden, structural progression, or long-term clinical outcomes in ACM. Accordingly, immunomodulatory therapy should remain investigational rather than standard-of-care pending prospective validation. Until such data are available, management of hot-phase ACM should prioritize established ACM care, including arrhythmic risk stratification, exercise modification, heart failure therapy when indicated, family screening, and ICD consideration according to consensus-based risk assessment.^4^^,^^18^^,^^19^^,^^21^^,^^22^^,^^56^

Accordingly, any consideration of anti-inflammatory or cytokine-directed therapy should be individualized, multidisciplinary, and preferably embedded within a research protocol or registry. The manuscript therefore frames IL-1 blockade and related strategies as biologically plausible but unproven, with standard ACM care remaining the foundation of management.

## Unresolved questions and future directions

Several caveats should temper interpretation of the inflammatory hot-phase paradigm. Much of the current evidence is derived from observational cohorts, case series, systematic reviews of heterogeneous reports, and translational models rather than prospective genotype-stratified studies.^7^^,^^16^^,^^17^^,^^19^^,^^20^^,^^27^^,^^38^^,^^48^ Definitions of hot phase vary across studies, and the temporal relationship among troponin elevation, edema, VAs, and later scar progression remains incompletely established. Therefore, inflammation should be framed as a plausible and potentially modifiable disease amplifier rather than a proven universal driver of ACM progression.

Despite growing recognition of inflammatory manifestations in ACM, multiple fundamental questions remain unresolved.

First, the mechanistic role of inflammation in ACM pathogenesis remains incompletely understood. Although inflammatory infiltrates, cytokine signaling, and edema-based imaging abnormalities have been observed in affected patients, it remains uncertain whether inflammation functions primarily as a driver of disease progression or as a secondary response to cardiomyocyte injury.

Second, the clinical utility of proposed inflammatory biomarkers—including anti-DSG2 autoantibodies and circulating inflammatory mediators—requires further validation before routine implementation in diagnostic or prognostic frameworks.^11^ Standardization of assay methodology and multicenter prospective validation will be necessary to determine reproducibility and clinical applicability.

Third, although advanced CMR techniques such as T2-mapping have improved recognition of myocardial edema, the optimal role of parametric imaging in longitudinal disease surveillance remains undefined. Future studies are needed to determine whether dynamic imaging markers correlate with disease progression, arrhythmic burden, or therapeutic response.^42^^,^^43^

Finally, whether targeted immunomodulation can alter the natural history of inflammatory-prone ACM phenotypes remains unknown. Randomized prospective investigations will be required to establish whether modulation of inflammatory pathways meaningfully reduces myocardial injury, fibrosis progression, or arrhythmic outcomes.

Addressing these questions will be essential to determine whether the inflammatory “hot phase” represents merely an epiphenomenon of myocardial injury or a mechanistically actionable therapeutic frontier in ACM.

Future studies should report hot-phase episodes using standardized variables: genotype, number and timing of episodes, peak troponin and assay type, ESR/CRP, CMR T1/T2/extracellular volume and LGE pattern, PET-CT findings when obtained, ambulatory/device rhythm burden during and after the episode, EMB/viral polymerase chain reaction when clinically performed, exercise exposure, immunomodulatory treatment, and subsequent scar progression, VT/VF, ICD therapy, heart failure, and transplantation. Without these harmonized data, it will remain difficult to determine whether inflammation independently modifies risk beyond genotype, LGE, ventricular function, and prior arrhythmia.^16^, ^17^, ^18^, ^19^, ^20^, ^21^^,^^26^^,^^27^^,^^42^^,^^43^^,^^48^^,^^56^, ^57^, ^58^

## Conclusions

The inflammatory hot phase of ACM represents an emerging paradigm in which episodic myocardial injury, edema, and immune activation may transiently amplify arrhythmic vulnerability on top of an inherited scar-prone substrate. For electrophysiologists, the key challenge is to distinguish isolated myocarditis from inflammatory-prone genetic ACM and to integrate dynamic inflammatory activity with established risk markers, including genotype, ventricular function, scar burden, arrhythmic history, family history, and exercise exposure.^4^^,^^22^^,^^48^^,^^52^, ^53^, ^54^, ^55^, ^56^, ^57^, ^58^ Although targeted immunomodulation remains investigational, inflammatory phenotyping may ultimately refine risk assessment by identifying periods of biologic and electrical instability that are not captured by static structural markers alone.

At present, the safest clinical framing is therefore not that hot phase mandates new treatment thresholds, but that it should trigger careful phenotyping: exclude mimics, identify inherited ACM when present, document dynamic arrhythmia burden, individualize exercise counseling, and reserve immunomodulatory therapy for selected situations in which myocarditis subtype, sarcoidosis, giant cell/eosinophilic disease, or immune-mediated inflammation is sufficiently supported.^18^, ^19^, ^20^, ^21^

## Funding support and author disclosures

The authors have reported that they have no relationships relevant to the contents of this paper to disclose.
